# Supplementary material for: Effect of Radiotherapy on the Right Ventricular Function in Lung Cancer Patients
Source: Cancers (Basel). 2024 May 23;16(11):1979. doi: 10.3390/cancers16111979 (PMC11171340; doi:10.3390/cancers16111979)
Supplement: Supplementary file 1 [file cancers-16-01979-s001.zip › Supplementary Table S1.pdf]

Supplementary Table S1. Echocardiographic parameters used to determine right ventricular systolic function.

Parameter	Abnormal value	Advantages	Limitations
TAPSE	<16 mm	Easy to measure<br>Reproducible<br>Less dependent on image quality	Expresses only the longitudinal shortening of the RV<br>Neglects contribution of RVOT to RV global function<br>Useless after cardiac surgery<br>Angle- and load-dependent
FAC	<35%	Reflects both longitudinal and radial contraction of the RV	Neglects contribution of RVOT to ejection<br>Requires good image quality<br>Load-dependent
IVA	<2.2 m/s	Relatively load-independent	Angle-dependent<br>Limited data available<br>Affected by age and heart rate
RV MPI using pulse wave Doppler	>0.40	Less affected by heart rate	Unreliable when RA pressure is elevated
RV MPI using tissue Doppler	>0.55	Less affected by heart rate	Unreliable when RA pressure is elevated
RV S’	<10 cm/s	Similar to TAPSE	Similar to TAPSE
RV LS (free wall)	> -22.5%	Angle-independent<br>Easy to measure	Neglects contribution of RVOT to RV global function

		Less sensitive to loading conditions	Good image quality required
RV LS	> -20%	Angle-independent<br>Easy to measure<br>Less sensitive to loading conditions	Neglects contribution of RVOT to RV global function<br>Good image quality required
3DRVEF	<44%	Includes contribution of RVOT to global RV function<br>Correlates with the RVEF obtained in the CMR<br>Reliable after cardiac surgery	Dependence on adequate image quality<br>Load-dependent<br>Time consuming<br>Off-line analysis and experience required

Abbreviations: 3D – three-dimensional; CMR – cardiac magnetic resonance; EF – ejection fraction; FAC – fractional area change; IVA – myocardial acceleration during the isovolumic phase of contraction; RA – right atrium; MPI – myocardial performance index; RV – right ventricle; RV LS – RV longitudinal strain; RVOT – RV outflow tract; TAPSE – tricuspid annular plane systolic excursion.
